# Reply to: Embracing the taxonomic and topological stability of phylogenomics

**DOI:** 10.1038/s41598-024-54487-x

**Published:** 2024-02-19

**Authors:** Hsin Lee, Kwen‑Shen Lee, Chia‑Hsin Hsu, Chen‑Wei Lee, Ching‑En Li, Jia‑Kang Wang, Chien‑Chia Tseng, Wei‑Jen Chen, Ching‑Chang Horng, Colby T. Ford, Andreas Kroh, Omri Bronstein, Hayate Tanaka, Tatsuo Oji, Jih‑Pai Lin, Daniel Janies

**Affiliations:** 1 National Museum of Marine Biology and Aquarium, Pingtung, 944401 Taiwan; 2 Department of Geosciences, National Taiwan University, Taipei, 106319 Taiwan; 3 Institute of Oceanography, National Taiwan University, Taipei, 10617 Taiwan; 4 Biology Department, National Museum of Natural Science, Taichung, 404023 Taiwan; 5 Tuple LLC, 2413 Commonwealth Ave, Charlotte, NC 28205 USA; 6 School of Data Science, University of North Carolina at Charlotte, 9201 University City Blvd, Charlotte, NC 28223 USA; 7 Department of Bioinformatics and Genomics, University of North Carolina at Charlotte, 9201 University City Blvd, Charlotte, NC 28223 USA; 8 Center for Computational Intelligence to Predict Health and Environmental Risks (CIPHER), University of North Carolina at Charlotte, 9201 University City Blvd, Charlotte, NC 28223 USA; 9 Department of Geology and Palaeontology, Natural History Museum Vienna, 1010 Vienna, Austria; 10 School of Zoology, Faculty of Life Sciences, Tel Aviv University, 6997801 Tel Aviv, Israel; 11 Steinhardt Museum of Natural History, Tel Aviv University, 6997801 Tel Aviv, Israel; 12 Department of Biological Sciences, Graduate School of Science, University of Tokyo, Tokyo, 113‑0033 Japan; 13 University Museum, Nagoya University, Furo‑cho, Nagoya, 464‑8601 Japan

**Keywords:** Evolutionary genetics, Biogeography

**replying to:** M. Koch; *Scientific Reports* 10.1038/s41598-024-54208-4 (2024).

## Introduction

We would like to re-emphasize our contribution on re-classification of Scutelliformes based on our new findings and appreciate the valid criticism raised by Koch^1^. In his reply to our work^2^, Koch^1^ criticized the use of a dataset based on four molecular markers (two mitochondrial loci: *Cox1* and *16S*, and two nuclear ones: *28S* and *H3*) for a reassessment of the classification of sand dollars. Koch^1^ pointed out the incongruence of certain deeper level splits in the tree published in our recent work^2^ with those based on their genome-scale datasets^3^. One of the taxonomic disagreements is the position of Laganiformes. In the original paper where Mongiardino Koch et al.^2^ proposed the new clade Luminacea, Laganiformes are represented by only two taxa that form a sister group to Scutelliformes. The position of Laganiformes appears more closely related to Clypeasteroida in our study^2^. In the classic morphological classification in Kroh and Smith^4^, these three clades are closely related. Luminacea, however, includes the fourth clade Cassiduloida, which is morphologically distinct from the other three sand dollar clades. In fact, there is no morphological synapomorphy for Luminacea; instead, it is based on molecular evidence. We tested the taxonomic stability within the clade Luminacea by increasing taxon sampling (from 15 taxa in Mongiardino Koch et al.^3^ to 25 taxa) with new sand dollar data from Taiwan and other countries.

Koch^1^ argue that the data presented in our study^2^ cannot fully resolve deep branching patterns of the major luminacean clades, and we fully agree with that. Perhaps, the best alternative is to present the inter-relationships among Cassiduloida, Laganiformes, Scutelliformes, and Clypeasteroida, as polytomies in our original study^2^. This is one of the reasons why we presented the novel classification (Lee et al.^2^) in a way that is restricted to the scutelliform clade of Luminacea which is well supported by both analyses. A full phylogenetic reassessment of Luminacea addressing deep splits within that clade was neither the subject of Lee et al.^2^ nor was any of the sister-group relationship contested by Koch^1^ used to propose a novel classification in conflict with previous results. Publication of data and results that are in conflict with previous analyses does not create a state of taxonomic instability or chaos. It is well established that gene trees differ from species trees^5^. As such, it comes as no big surprise that the number of markers applied and taxonomic sampling effort affect the results of individual analyses, and deeper level splits in particular. We argue that for the progress of science it is necessary to report results even if they are in conflict with other datasets. From this point we can discuss potential reasons for the observed relationships and what the next steps are to improve our understanding of the relationships.

The main concern expressed by Koch^1^, namely the sister-group relationship between laganids and clypeasteroids as outlined in the tree of our work^2^, was only briefly addressed in that study. We did not consider the matter further in the conclusions or novel classification, because the respective nodes were poorly supported in the reconstructed tree. Moreover, the authors were well aware (and in part involved) in the genomic-scale studies of Mongiardino Koch et al.^3,6^. We regret that this has not been expressed more clearly in our work^2^ and acknowledge Koch^1^ for rectifying this omission. The main finding of our study^2^, namely, the relationship of the three main clades composing the Scutelliformes (Astriclypeoidea, Mellitoidea, and Taiwanasteroidea) remains valid. These clades, corresponding to the so named superfamilies, are well supported also in the trees provided by Koch^1^ in Figure S2.C and S2.D and S2.A and S2.B, if the incorrectly placed spatangoids are ignored in the latter two trees.
